# Hæmaturia : The Diagnosis of Its Cause

**Published:** 1891-09-26

**Authors:** 


### SURGERY.

### HÆMATURIA: THE DIAGNOSIS OF ITS CAUSE.

The diagnosis of the cause of hæmaturia is of great interest both to the physician and the surgeon. It is one of those affections which do not suit the specialist, but requires knowledge both of medicine and surgery to diagnose its cause, with a view to successful treatment.

The difficulties which beset us in our endeavour to find out the cause of hæmaturia sometimes are well illustrated by the following case lately under our care: A middle-aged man, apparently in perfectly health, begins to pass urine deeply coloured red with blood. This continues day after day for a week without any other symptoms whatever, and then wholly and suddenly ceases, so that no blood corpuscles are even seen with the microscope. There was no pain anywhere, even on exercise, no undue frequency in micturition, and no vesical or lumbar tenderness, nor had the patient been out of this country, and run any risk of getting the hilharzia into his urinary organs. The first thing we think of when a patient passes blood in his urine, day after day, or perhaps month after month, without any other symptoms, is villous tumour of the bladder. But in this disease there is a very interesting peculiarity about the hæmaturia; it comes at the end of micturition. When the patient begins the urine may be quite clean, but as the bladder contracts on the growth it bleeds, and the urine passed towards the end of the act becomes coloured with blood. This character of the hæmaturia was, however, wholly wanting in this case. We have known a person treated for years for constant hæmaturia by remedies supposed to influence his liver, who, all the time, was suffering from a villous tumour of his bladder which then gave rise to no other symptoms. It was eventually diagnosed and removed, but, unfortunately, its base was by that time so large, with a fatal result. But in the diagnosis of villous growth in the bladder we must not rely on the character of the bleeding only; we must try and find fragments of villous growth in the urine; and it has even been recommended to try and get bits of the growth by the use of the lithotrity evacuating bottle and catheter, the strong suction tending to detach minute filaments of the growth. Malignant growth in the bladder will also cause hæmaturia, but not generally apart from other urinary symptoms, as is so often the case with simple villous tumour. If the probability is strong that a villous growth is the cause of serious hæmaturia we may be justified in exploring the bladder with a lithotrite by which means the diagnosis has been made, or even by an exploratory opening into the bladder, being prepared to remove this growth if found suitable for removal.

But there are cases where persistent hæmaturia without any other symptoms, does not seem due to villous growth in the bladder, as in the case referred to. In some of these cases a calculus has been discovered fixed in a calyx of the kidney, and hence we must not look on the absence of renal pain and tenderness and other urinary symptoms as absolutely contra-indicating such a diagnosis. Indeed, we believe this to be the cause of the hæmaturia in this case. On one occasion on which we examined the urine sediment microscopically we found besides innumerable red corpuscles a well-formed, but very large blood cast, evidently formed in a very large tube, and twice after we found fragments of such casts. This, of course, settled the question as to its renal origin, and the dye of the cast suggested rather the reflux of blood from the pelvis into a collecting tube than hæmorrhage into the smaller tubes. Moreover, there were constantly present uric acid crystals, and often oxalate crystals as well, and in many samples numerous pus cells were seen. All these things seemed to us to suggest renal calculus; but the bleeding suddenly ceased, and the patient is about again. Could a calculus have slipped into the orifice of the ureter and cut off the urinary excretion from that kidney without an attack of renal pain? If so this may account for the sudden cessation of the hæmaturia. The urine passed then was not only absolutely free from blood, but contained no albumen, which excluded any inflammatory origin of the bleeding. We have not seen him since, so cannot say if any signs of lychonephidris have developed.

But Hilton Fagge says in his text-book of medicine, that he has seen two or three cases like this one, in which hæmaturia has been present for days, and then wholly subsided, and it has been quite impossible to say what the hæmaturia was due to. We remember a case in the Metropolitan Hospital where we studied, in which no cause for persistent hæmaturia could be discovered, until it was ascertained that the man was a "bleeder." But this is, indeed, a rare cause.

When renal hæmorrhage is not great, the blood gives the urine the characteristic "smoky tint" instead of the bright red; but on the other hand, renal hæmaturia may be quite a bright red colour when profuse. To distinguish it from vesical hæmorrhage, we must examine the urine for casts, and as the blood disappears, for albumen; but we may have to be guided very much by other symptoms present. For instance, if there is lumbar pain of a dull aching character, with, perhaps, only slight œdema about the eyelids, and sickness and slight fever, it is probably the result of acute nephritis, or again, certain drugs, such as cantharides and turpentine may cause it. Here it is in the renal tissue, and casts will be present, showing changes in the epithelial cells, and small blood casts. Renal hæmorrhage may also be due to malignant growths of the kidney, and here palpation, perhaps under an anæsthetic, may decide as to its origin. If, on the other hand, a patient passing blood with his urine, complains of sharp pains, or sometimes dull aching pain in one lumbar region chiefly, but often shooting down into the groin on that side, and in men causing retraction of the testicle—pain increased by a violent movement of the back, or jolting in a cart—then (especially if we find uric acid crystals in the urine) we may diagnose renal calculus as its cause.

We have already discussed the diagnosis of tumours of the bladder as a cause of hæmaturia. The hæmaturia of stone is generally accompanied by the other symptoms of stone, and pus, as well as blood, is found in the urine. In elderly men with enlarged prostates, the prostatic veins often bleed into the bladder.

These are some of the more important causes of hæmaturia. We cannot discuss them all, nor is it needful to do more than refer to injuries of the kidney, the diagnosis of the cause here is obvious. Hæmorrhage from the urethra must not altogether be forgotten, but we cannot consider it here. Our chief difficulty is in cases where hæmaturia is profuse and continuous, and where no other guiding symptoms are present to point out the possible causes of this condition, and the methods to be employed in their diagnosis is the chief object of this paper.

